# Poor clinical outcomes among hospitalized obese patients with COVID-19 are related to inflammation and not respiratory mechanics

**DOI:** 10.2478/jccm-2025-0012

**Published:** 2025-04-30

**Authors:** Jordan N Edwards, Tomas Ganz, Elizabeta Nemeth, Emily J Martin, Nicholas J Jackson, Airie Kim

**Affiliations:** David Geffen School of Medicine, University of California, Los Angeles, CA, USA

**Keywords:** COVID-19, obesity, inflammation, hospital outcomes, respiratory mechanics

## Abstract

**Introduction:**

The coronavirus disease 2019 (COVID-19) has infected millions of people worldwide resulting in high morbidity and mortality. Obesity is known to cause metabolic derangements and precipitate worse outcomes from viral pneumonia, potentially secondary to increased inflammation and/or altered respiratory mechanics.

**Aim of the Study:**

Our study’s aim was to examine the relationships among BMI, systemic inflammation, and respiratory mechanics in determining clinical outcomes.

**Materials and Methods:**

This retrospective, observational cohort study included 199 adult patients with confirmed COVID-19 who were hospitalized at a quaternary-referral academic health system. Data were manually extracted from electronic medical records, including baseline demographics and clinical profiles, inflammatory markers, measures of respiratory mechanics, and clinical outcomes. We used the rank-sum test to compare the distributions of BMI and inflammatory markers between those with and without specific clinical outcomes, and the Pearson correlation to measure the correlations between BMI and inflammatory markers or respiratory mechanics.

**Results:**

Higher BMI was associated with worse clinical outcomes, including the need for Intensive Care Unit (ICU) admission, invasive mechanical ventilation (IMV), neuromuscular blockade, and prone positioning, particularly in male patients. Inflammation, as measured by C-reactive protein, lactate dehydrogenase (LDH), ferritin, and D-Dimer, was also increased in both male and female patients who required ICU admission, IMV, neuromuscular blockade, and prone positioning. However, only male patients had a positive correlation of LDH and D-Dimer levels with BMI. There was no correlation between BMI and respiratory mechanics, as measured by static compliance and the response to prone positioning.

**Conclusions:**

Our findings suggest that the metabolic dysfunction and systemic inflammation seen in obesity, and not dysfunctional respiratory physiology, drive the negative clinical outcomes seen in this cohort of hospitalized COVID-19 patients.

## Introduction

In December 2019, outbreaks of pneumonia from an unknown organism were first recognized in Wuhan, China. A novel coronavirus, Severe Acute Respiratory Syndrome Coronavirus 2 (SARS-CoV-2), was quickly identified as the microbe causing the corona-virus disease 2019 (COVID-19). Since then, there have been over 200,000,000 confirmed cases and more than 4,500,000 deaths worldwide [1]. COVID-19 presents with a wide spectrum of disease, from asymptomatic infection to severe acute respiratory distress syndrome (ARDS) and death.

Obesity, defined as a body mass index (BMI) ≥ 30 kg/m^2^, is also a serious public health concern [2] that affects more than one-third of adults and 17% of children in the United States [3]. Adipose tissue plays a critical and beneficial role in metabolic homeostasis, but excess adiposity is associated with increased morbidity and mortality. Central obesity is an important feature of metabolic syndrome, a condition that manifests in chronic inflammation, dyslipidemia, insulin resistance, hyperglycemia, hypertension, and ultimately worsened cardiovascular outcomes [4]. Approximately 90% of individuals with type 2 diabetes are overweight or obese [5], and the risk of diabetes increases almost 5% with every kg increase in weight [6].

Obesity consistently ranks among the highest underlying comorbidities in patients hospitalized with COVID-19 [7], and is associated with worse outcomes including hospitalization, Intensive Care Unit (ICU) admission, mechanical ventilation, and mortality [8]. Studies have suggested that central obesity-associated inflammation may mediate the worse COVID-19 outcomes seen in these patients [9]. In addition to the standard laboratory indicators of inflammation, metabolic parameters such as BMI and waist and hip circumference could be helpful for risk stratification [10]. There has also been speculation that abnormal respiratory mechanics and ventilation/perfusion mismatch could contribute to adverse outcomes in patients with excess adiposity [8]. However, the potential contribution of the mechanical consequences of obesity to worse outcomes in COVID-19 remains incompletely understood. In this study, we examined the role of obesity in COVID-19 severity at a quaternary care academic medical center composed of two hospitals, analyzing the relationships among inflammation, mechanical ventilatory parameters, and obesity in male and female COVID-19 patients.

## Methods

### Setting and study design

**Fig. 1.**
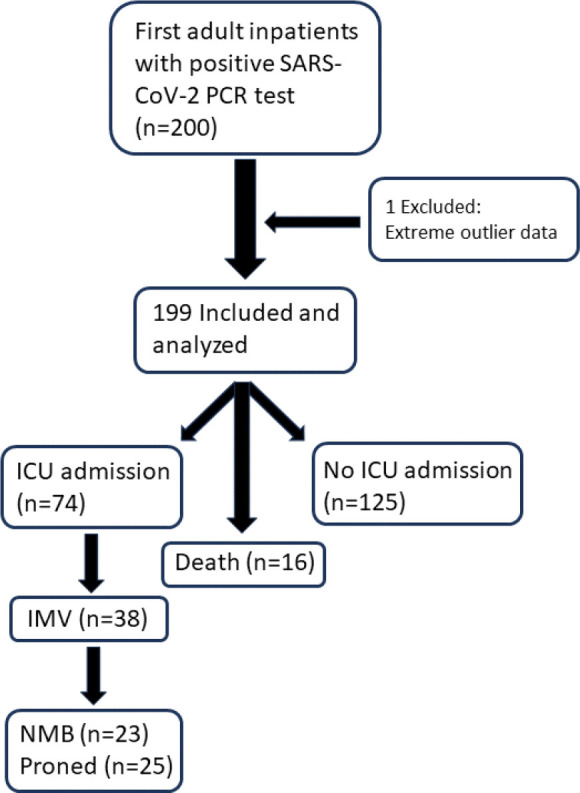
Flowchart of study subject selection and clinical outcomes. ICU = Intensive Care Unit; IMV = invasive mechanical ventilation; NMB = neuromuscular blockade.

This is a retrospective observational cohort study conducted from December 13, 2019 to June 26, 2020, which was approved by the UCLA (University of California, Los Angeles) Institutional Review Board with waiver of informed consent. The UCLA Health system includes two quaternary-care academic medical centers, Ronald Reagan-UCLA Medical Center (RR-UCLA) and Santa Monica-UCLA Medical Center (SM-UCLA), which have 154 ICU beds and 38 ICU beds, respectively. The study included 199 adult hospitalized patients at RR-UCLA and SM-UCLA with positive SARS-CoV-2 PCR testing from either nasal swab or mini-bronchoalveolar lavage (BAL) testing, and their data were retrospectively extracted from the electronic health record (Figure 1).

### Clinical protocols

As this was an observational study, all clinical management was left to the discretion of the clinical teams. A hospital pandemic response team made up of intensivists and infectious disease physicians had generated COVID-19 clinical guidelines, which informed patient management decisions. These guidelines incorporated established best critical care practices for acute respiratory distress syndrome (ARDS), including low tidal volume ventilation, early consideration of prone ventilation for patients with PaO_2_:FiO_2_ (P:F) ratios ≤ 150, daily awakening trials or light sedation, and conservative fluid management. In addition, the clinical guidelines also recommended early endotracheal intubation for COVID-19 patients with quickly escalating supplemental oxygen requirements.

### Data collection

Baseline demographic data, medical comorbidities, and body mass index (BMI) were collected from the electronic medical record. Inflammatory markers, including C-reactive protein (CRP), lactate dehydrogenase (LDH), ferritin, and D-Dimer were obtained as peak values from the hospitalization period.

The following outcome data were collected: admission to the ICU, invasive mechanical ventilation (IMV), in-hospital mortality (during the first 60 days of hospitalization), neuromuscular blockade (NMB), and prone positioning. For patients who had invasive mechanical ventilation during their hospitalization, we collected maximum and minimum static compliance values as recorded by the respiratory therapist. For patients who underwent proning, we quantified their P:F response to proning as the percent improvement in P:F ratio as calculated by arterial blood gas PaO_2_ and the ventilator FiO_2_ setting. In short, P:F response to proning = (P:F ratio after proning – P:F ratio before proning) / P:F ratio before proning X 100%.

### Statistical methods

We summarized the baseline characteristics of the cohort by obesity status using median with interquartile range (for age) and relative frequency (for categorical variables). Differences in baseline characteristics between obese and non-obese participants were assessed using a Wilcoxon-Mann-Whitney rank-sum test (for age) and two-sample test of proportions (for categorical variables). We then compared the distributions of BMI and measures of systemic inflammation (CRP, LDH, Ferritin, D-Dimer) between those with and without key clinical outcomes (ICU admission, IMV, death, NMB, proning) using the rank-sum test. Differences were visualized using box-plots. Sensitivity analyses examined associations after removal of severely under-weight patients (BMI < 18.5) and in analyses stratified by sex and age (<60 years). Within-sex BMI associations with measures of systemic inflammation and respiratory mechanics were examined using Pearson correlations. SigmaPlot version 12.5 (Systat Software, San Jose, CA) was used for all statistical analyses.

## Results

### Characteristics of our patient cohort

We evaluated the first 199 unique admissions to RR-UCLA and SM-UCLA with confirmed COVID-19 infection, diagnosed from December 13, 2019 to June 26, 2020 (Table 1). The median age was 63 years (IQR 45–77), and subjects were predominantly male (59%). The self-identified cohort ethnicities were 39% White, 32% Latinx, 10% Black, 9% Asian, and 11% Other. The majority (92%) had a known comorbidity on admission, with the most frequent comorbidities being obesity (33%), hypertension (55%), hyperlipidemia (38%), diabetes (31%), chronic kidney disease (23%), and coronary artery disease (22%). Obesity had a significant association with younger age, White or Latinx race/ethnicity, absence of other comorbidities, and a diagnosis of obstructive sleep apnea (Table 1).

**Table 1.** Demographics and clinical characteristics of cohort

	**Total (N = 199)**	**Non-obese (N = 133)**	**Obese (N = 66)**	**p-value**
Age				
Median (IQR)	63 (45, 77)	69 (51, 82)	53 (41, 65)	**<0.001**

Sex				
Male	118 (59%)	80 (60%)	38 (58%)	0.845
Female	81 (41%)	53 (40%)	28 (42%)	0.845

Race/Ethnicity				
Black	19 (10%)	14 (11%)	5 (7%)	0.681
White	78 (39%)	59 (44%)	19 (29%)	**0.049**
** **Asian	17 (9%)	13 (10%)	4 (6%)	0.540
Latinx	63 (32%)	31 (23%)	32 (48%)	**<0.001**
** **Other	22 (11%)	16 (12%)	6 (9%)	0.702

Comorbidities				
No PMHx	16 (8%)	15 (11%)	1 (2%)	**0.035**
Diabetes w/end-organ damage*	20 (10%)	12 (9%)	8 (12%)	0.664
Diabetes w/o end-organ damage*	41 (21%)	26 (20%)	15 (23%)	0.737
Hypertension	109 (55%)	75 (56%)	34 (52%)	0.618
Hyperlipidemia	76 (38%)	53 (40%)	23 (35%)	0.597
CKD	45 (23%)	27 (20%)	18 (27%)	0.354
COPD	21 (11%)	16 (12%)	5 (8%)	0.473
CAD	44 (22%)	30 (23%)	14 (21%)	0.973
Cancer	4 (2%)	4 (3%)	0 (0%)	0.375
Asthma	28 (14%)	18 (14%)	10 (15%)	0.926
Atrial arrhythmia	19 (10%)	14 (11%)	5 (8%)	0.681
Myocardial infarction	9 (5%)	4 (3%)	5 (8%)	0.272
Congestive heart failure (symptomatic)	15 (8%)	10 (8%)	5 (8%)	0.999
Obstructive sleep apnea	16 (8%)	4 (3%)	12 (18%)	**<0.001**

Age is reported as median (IQR), and all other data are reported as n (%). The p-value for age was obtained by Wilcoxon-Mann-Whitney rank-sum test. For non-age categories, p-values were obtained using a two-sample test of proportion. IQR: interquartile range; PMHx: Past medical history; CKD: chronic kidney disease; COPD: chronic obstructive pulmonary disease; CAD: coronary artery disease.

* retinopathy, neuropathy, nephropathy

### Poor clinical outcomes in hospitalized COVID-19 patients are associated with higher BMIs

Our study showed that patients with worse clinical outcomes had higher BMIs (Online supplementary material - Figure S1A), including patients who required ICU care (median BMI 28.2 kg/m^2^ vs 26.2, P = 0.031), patients on IMV (median BMI 30.9 kg/m^2^ vs 26.2; P = 0.001), patients who required neuromuscular blockade to manage severe hypoxemia during mechanical ventilation (median BMI 31.0 kg/m^2^ vs 26.3, P <0.001), and/or patients placed in prone positioning to improve ventilation/perfusion (V/Q) mismatch (median BMI 32.3 kg/m^2^ vs 26.3, P <0.001). Interestingly, patients who died during the hospitalization had similar BMIs to those who did not. These findings remained true after exclusion of the seven underweight (BMI <18.5 kg/m^2^) patients in the cohort (Online supplementary material - Figure S1B). We found similar findings in the sex stratified analyses (Online supplementary material - Figure S1C), though reliable differences were mostly found within the male strata potentially due to the smaller sample size of females. However, with the exception of mortality outcome, each worse outcome in females was associated with a higher BMI.

Outcomes were then analyzed after excluding patients ≥60 years old, as advanced age itself is a very strong predictor of worse COVID-19 outcomes (Online supplementary material - Figure S2) [11]. In this younger cohort, patients who required IMV and/or proning had significantly higher BMIs than those who did not, with median BMIs of 34.0 kg/m^2^ vs 27.7 (IMV) and 35.6 kg/m^2^ vs 27.9 (proning), respectively (Online supplementary material - Figure S2A). While the parameters of ICU admission, neuromuscular blockade, and death were not reliable at a p<0.05 level, the median BMIs were still higher for the worse outcome in each category.

When males and females under 60 years of age were analyzed separately, results were similar to the combined sex analyses, though with only the male subset showing reliable results (Online supplementary material - Figure S2B). The median BMIs for male patients who underwent ICU admission, intubation, paralysis, proning, and/or death were all higher than those who did not, either with statistical significance or a strong trend. The median BMI for young males requiring IMV was higher at 34.8 kg/m^2^, as opposed to 28.5 kg/m^2^ in the non-intubated group (P = 0.007). Similarly, the BMIs for young males requiring neuromuscular blockade or proning were higher as compared to those who did not, at 35.6 kg/m^2^ vs 28.7 (P=0.015) and 36.0 kg/m^2^ vs 29.2 (P=0.002), respectively. Again, young females in all outcome categories did not have median BMIs that were significantly different between positive and negative outcomes (Online supplementary material - Figure S2B).

### Poor clinical outcomes in hospitalized COVID-19 patients are associated with increased systemic inflammation

We observed that median peak levels of multiple inflammatory markers were higher in patients with worse outcomes from COVID-19 (Online supplementary material - Figure S3). The median CRP (Figure S3A), LDH (Figure S3B), ferritin (Figure S3C), and D-Dimer (Figure S3D) levels were all higher in patients who required ICU admission, IMV, NMB, proning, and/or did not survive the hospitalization. All inflammatory measure associations with clinical outcomes (aside from ferritin and survival) were statistically significant, showing higher levels of inflammation with worse clinical outcome. Similar results were found in sex stratified analyses such that the inflammatory parameters were persistently increased in patients with poor outcomes, with the exceptions of ferritin values for mortality and LDH values for female mortality (Online supplementary material Figures S4A-D). Potential contributors to this lack of significant difference is the non-inflammatory determinant of ferritin levels, namely iron status, and the small sample size of female subjects who had in-hospital deaths.

### BMI values correlate with increased systemic inflammation in hospitalized male COVID-19 patients

**Fig. 2.**
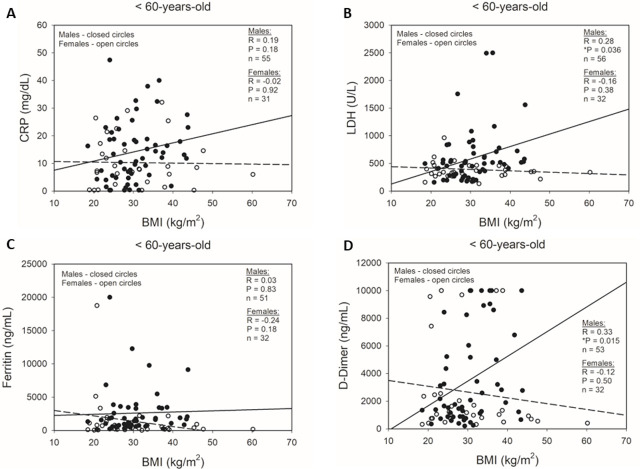
Correlations between BMI and inflammatory markers in <60-year-old male and female patients during COVID-19 hospitalization. Simple scatter plots of male and female patients with regression analyses of correlations between BMI and peak inpatient (A) CRP, (B) LDH, (C) ferritin, and (D) D-Dimer. ●: male subjects; ○: female subjects; solid regression line: male subjects; dashed regression line: female subjects. P-values calculated by Pearson Product Moment Correlation. *P<0.05.

After determining that increased obesity and systemic inflammation were both associated with disease severity in hospitalized COVID-19 patients, we examined whether there was a positive correlation between inflammatory lab values and BMI in male and female patients (Online supplementary material Figures S5). In males, we found that both LDH (Figure S5B) and D-Dimer (Figure S5D) levels had statistically significant positive correlations with BMI values. This correlation was not found with CRP (Figure S5A) or ferritin (Figure S5C). In females, none of the inflammatory parameters had significant correlations with the BMI values. This pattern of significant correlations of LDH and D-Dimer with BMI in males only was persistent when only subjects <60-years-old were included in the analysis (Figure 2). Interestingly, both the positive correlations of LDH and D-Dimer versus BMI had stronger correlations in the younger male subjects as compared to the all-age group. In males <60-years-old, both LDH (Figure 2B) and D-Dimer (Figure 2D) levels had statistically significant positive correlations (LDH: Rho = 0.28, P = 0.04; D-Dimer: Rho = 0.33, P = 0.015) with BMI measures.

### Respiratory mechanics may not be a significant contributor to the role of obesity in worse COVID-19 outcomes

**Fig. 3.**
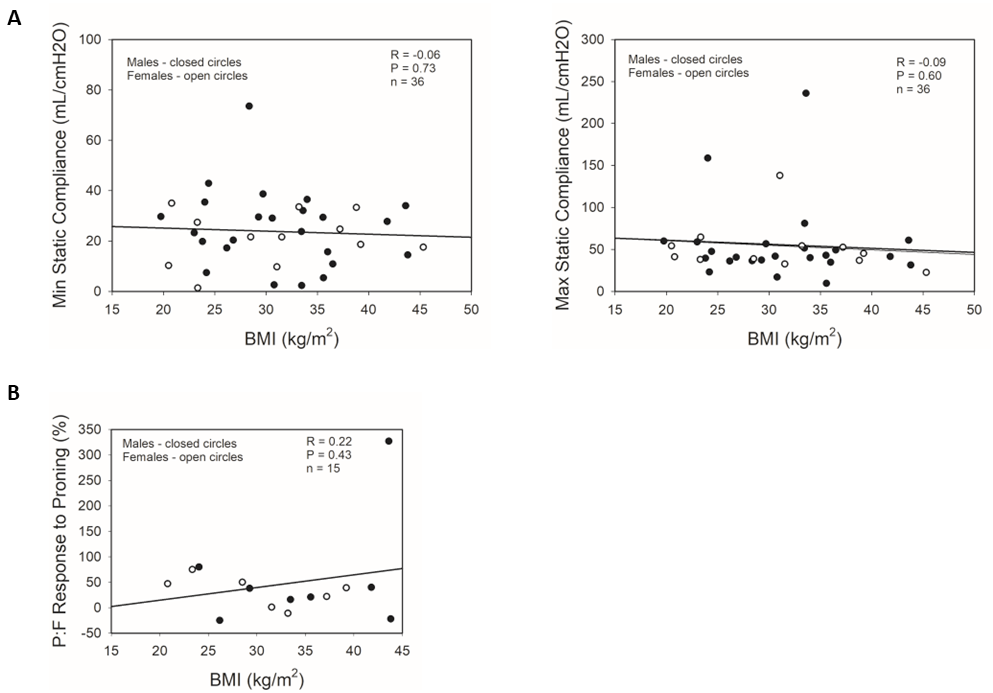
Correlations between BMI and respiratory physiology parameters in male and female intubated COVID-19 patients. Simple scatter plots of male and female patients with regression analyses of correlations between BMI and (A) minima and maxima static compliances during IMV, and (B) P:F responses to proning in male and female patients. ●: male subjects; ○: female subjects; regression lines represent combined male and female data. P:F response to proning = (P:F ratio after proning – P:F ratio before proning) / P:F ratio before proning X 100%. P-values calculated by Pearson Product Moment Correlation. IMV: invasive mechanical ventilation; P:F = ratio of partial pressure of arterial oxygen (PaO_2_): fraction of inspired oxygen (FiO_2_) of mechanical ventilator.

In order to investigate whether respiratory mechanics plays a role in the worse clinical outcomes of obese patients, we evaluated the relationship between lung static compliance, as measured by the ventilator, and the patient’s BMI. We found no significant correlation between BMI and maximum or minimum static compliance levels in ventilated patients (Minimum Rho = −0.06, P = 0.73, N = 36; Maximum Rho = −0.09, P = 0.60, N = 36) (Figure 3A). We also analyzed the potential role of body habitus in V/Q mismatch by calculating each patient’s response to proning, as measured by P:F ratio improvements, and correlating the response with the patient’s BMI (Figure 3B). There was no significant relationship between BMI and P:F response after proning for our patients, either in aggregate (Rho = 0.22, P = 0.43, N = 15) or separated by sex (males: Rho = −0.53, P = 0.23; females: Rho = 0.34, P = 0.40). In total, these parameters of lung mechanics and physiology do not indicate a significant role in mediating the effects of BMI status on COVID-19 in-hospital outcomes.

## Discussion

Since the beginning of the COVID-19 pandemic, numerous studies have attempted to identify the most significant risk factors for severe COVID-19 as defined by multiple parameters including hospitalization, ICU admission, IMV, and mortality [12]. Obesity was quickly identified as a significant risk factor, as findings from an early study in Shenzhen, China showed that obese patients had a 3.4-fold odds of developing severe pneumonia from COVID-19 than non-obese patients [13]. Similar observations were made in New York City, where a retrospective case series from two hospitals showed that patients who received IMV were more likely to be obese and had increased inflammatory markers as compared to those who did not undergo intubation [14]. A subsequent study from a tertiary medical center in the Bronx, New York, showed that severe obesity (defined by BMI ≥35kg/m^2^) and increasing age were both independent risk factors for in-hospital mortality [15]. Together, these studies indicated that obesity was an important risk factor for developing severe disease from SARS-CoV-2 infection.

Our UCLA study was a retrospective analysis of 199 hospitalized COVID-19 patients at two academic medical centers. 33% of the patients were obese, as defined by a BMI ≥30kg/m^2^. We found that patients with severe disease who required ICU admission, mechanical ventilation, prone positioning, and/or neuromuscular blockade had significantly higher median BMIs than those patients who did not require these interventions. As being underweight is also a risk factor for increased mortality and worse outcomes in multiple disease processes [16,17], we confirmed that these findings persisted with or without the inclusion of underweight subjects in the analysis. When separating subject groups by male or female sex, we found that this association was statistically stronger in males in both the all-age and <60-year-old groups. While the differences in statistical strengths between males and females may be partially a reflection of sample size, the consistency of this stronger association with multiple clinical outcomes suggests that males’ BMIs are more strongly linked to COVID-19 in-hospital outcomes than females’ BMIs.

A similar association between obesity and worse outcomes in viral pneumonia has been reported previously with influenza A virus. One case-cohort study evaluated hospitalizations and deaths from the 2009 influenza A (H1N1) pandemic and found that obesity and morbid obesity were associated with increased odds (OR 3.1 and 7.6, respectively) of death [18]. Case studies of severe Influenza A virus infections in obese patients have illustrated extensive viral replication and prolonged viral shedding [19]. Despite the common clinical observation of worse influenza A outcomes with obesity, the underlying mechanisms are incompletely understood and likely multifactorial. One commonly proposed mechanism of obesity-related immune dysfunction is the underlying chronic inflammatory state of obesity, with increased levels of inflammatory cytokines including TNFα, IL6, and leptin [20]. The resulting inflammatory milieu can induce endoplasmic reticulum stress, production of reactive oxygen species, and oxidative stress, resulting in dysregulation of the innate immune response to viral infection. When infected with influenza A, the adipose tissue of two mouse models of obesity produced increased levels of MCP-1, TNFα, IL-6, and TGFβ [19]. Additional animal studies showed that obese mice infected with influenza A had a 6.6-fold greater mortality rate as compared to lean mice, with greater lung pathology and marked differences in the production of antiviral and proinflammatory cytokines [21]. Human in vitro studies of CD4+ and CD8+ T cells from obese and overweight adults expressed lower levels of CD28, CD40L, CD69, and IL-12R when stimulated with H1N1, suggesting that T cell dysfunction contributes to the increased morbidity and mortality from H1N1 infection in these patients [22].

Given the likely contribution of immune dysregulation to the worse outcomes seen in obese patients with COVID-19, we hypothesized that the degree of obesity correlated with the severity of inflammation. We confirmed the observations in existing literature that worse outcomes are associated with increases in inflammatory markers, including CRP, LDH, ferritin, and D-Dimer. When correlating BMI with these inflammatory lab values, we found correlation with some of the inflammatory parameters in males only, with no correlation in female subjects. We speculate that this sex difference could be related to the increased visceral adiposity in males [23], as visceral adipose tissue (VAT) has been shown to be more inflammogenic than subcutaneous adipose tissue and associated with metabolic and cardiovascular disorders [24,25]. Multiple clinical studies have investigated the relationship between visceral adiposity and severe COVID-19 disease [26,27], and found that increased VAT as quantified by CT scan was independently associated with ICU admission. Thus, the differential associations between BMI and inflammation in males versus females could be related to the sex differences in visceral adiposity.

In addition to the associated innate immunity derangements, obesity also has a significant impact on the mechanics and physiology of respiration. Excess deposition of adipose tissue in the mediastinum and abdomen causes mechanical and anatomical changes that lead to reduced compliance of the lungs and chest wall and decreased airway caliber [28]. These alterations also increase ventilation-perfusion mismatch by preferentially distributing the resting tidal volume to the upper lung zones whereas perfusion distribution is preferentially to the lower lung zones, leading to reduced PaO2 levels and an increased alveolar-arterial gradient [29,30]. In order to assess the potential contribution of altered lung mechanics to the worse outcomes in obese patients, we evaluated whether lung static compliance and/or P:F response to proning had any correlation with BMI in patients on invasive mechanical ventilation. We found no significant correlation between BMI and either respiratory parameter, indicating no obvious contribution of lung mechanics to the worse outcomes with higher BMIs. This outcome is not surprising as some studies have shown that obese patients can have improved outcomes with ARDS [31,32] and/or bacterial pneumonia [33,34], the so-called “obesity paradox”. The altered respiratory mechanics of obese patients would presumably also apply to these pathologies that demonstrate the “obesity paradox,” suggesting that systemic or non-anatomic factors play more significant roles than respiratory physiology in the response to lung injury and infectious pneumonias. Of note, an important limitation in our study was that these respiratory measurements were only obtained in intubated patients, greatly restricting the number of subjects and only including the most critically ill patients. Additional studies are required to elucidate the specific roles of altered pulmonary physiology in the response to viral pneumonia.

An important difference from other studies is the lack of clear association between obesity and mortality in our COVID-19 patients [35,36], although there is a trend of increased BMI with mortality when we exclude patients over 60-years-old. As age itself is a strong risk factor for severe COVID-19 disease and is associated with multiple other comorbidities, the exclusion of older subjects may allow the detection of an association between BMI and mortality. Another likely contributor is the small number of subjects that died during the study, limiting the statistical power of our analysis.

## Conclusion

In summary, the current article analyzes the hospital course, outcomes and characteristics of 199 hospitalized COVID-19 patients treated in our 2-hospital system. Our study confirms the findings in other studies that showed significant associations of BMI and inflammation with the severity of disease. We have also demonstrated that there may be sex-specific differences in the relationship between BMI and inflammation, potentially secondary to the increased visceral adiposity seen in males versus females. Finally, our study found no significant correlation between BMI and lung physiology parameters, indicating that restrictive lung physiology and V/Q mismatch may not be significant contributors to the worse outcomes seen in obese patients with COVID-19. Future investigations are necessary to further elucidate the relationships among obesity, sex, inflammation, and lung mechanics that lead to worse outcomes in COVID-19 and other viral penumonias.
